# Genetic Association of Hematopoietic Stem Cell Transplantation Outcome beyond Histocompatibility Genes

**DOI:** 10.3389/fimmu.2017.00380

**Published:** 2017-04-03

**Authors:** Rihab Gam, Pranali Shah, Rachel E. Crossland, Jean Norden, Anne M. Dickinson, Ralf Dressel

**Affiliations:** ^1^Hematological Sciences, Institute of Cellular Medicine, Newcastle University, Newcastle upon Tyne, UK; ^2^Institute of Cellular and Molecular Immunology, University Medical Centre Göttingen, Göttingen, Germany

**Keywords:** gene expression profiling, regulatory networks, non-human leukocyte antigen single-nucleotide polymorphisms, microRNAs, forkheadbox protein 3, miR-155, miR-146a, MICA

## Abstract

The outcome of hematopoietic stem cell transplantation (HSCT) is controlled by genetic factors among which the leukocyte antigen human leukocyte antigen (HLA) matching is most important. In addition, minor histocompatibility antigens and non-HLA gene polymorphisms in genes controlling immune responses are known to contribute to the risks associated with HSCT. Besides single-nucleotide polymorphisms (SNPs) in protein coding genes, SNPs in regulatory elements such as microRNAs (miRNAs) contribute to these genetic risks. However, genetic risks require for their realization the expression of the respective gene or miRNA. Thus, gene and miRNA expression studies may help to identify genes and SNPs that indeed affect the outcome of HSCT. In this review, we summarize gene expression profiling studies that were performed in recent years in both patients and animal models to identify genes regulated during HSCT. We discuss SNP–mRNA–miRNA regulatory networks and their contribution to the risks associated with HSCT in specific examples, including forkheadbox protein 3 and regulatory T cells, the role of the miR-155 and miR-146a regulatory network for graft-versus-host disease, and the function of MICA and its receptor NKG2D for the outcome of HSCT. These examples demonstrate how SNPs affect expression or function of proteins that modulate the alloimmune response and influence the outcome of HSCT. Specific miRNAs targeting these genes and directly affecting expression of mRNAs are identified. It might be valuable in the future to determine SNPs and to analyze miRNA and mRNA expression in parallel in cohorts of HSCT patients to further elucidate genetic risks of HSCT.

## Introduction

A considerable proportion of the risk of adverse outcome after hematopoietic stem cell transplantation (HSCT) is genetically determined and can be attributed to various factors including human leukocyte antigen (HLA) matching, killer-immunoglobulin-like receptor matching, minor histocompatibility antigens (miHAg), and non-HLA gene polymorphisms. Outcomes such as acute and chronic graft-versus-host disease (aGvHD and cGvHD), relapse, and survival have been shown to be modified by functionally relevant polymorphisms in non-HLA genes that are involved in immune responses (1, 2). Such functional polymorphisms are complicated to pinpoint among other polymorphisms localized near these genes that have no direct effects on gene function. Reliable identification of polymorphisms that result in differences in gene expression or protein function and affect the outcome of HSCT is challenging in view of the complexity of the human genome (3).

**Figure 1:**
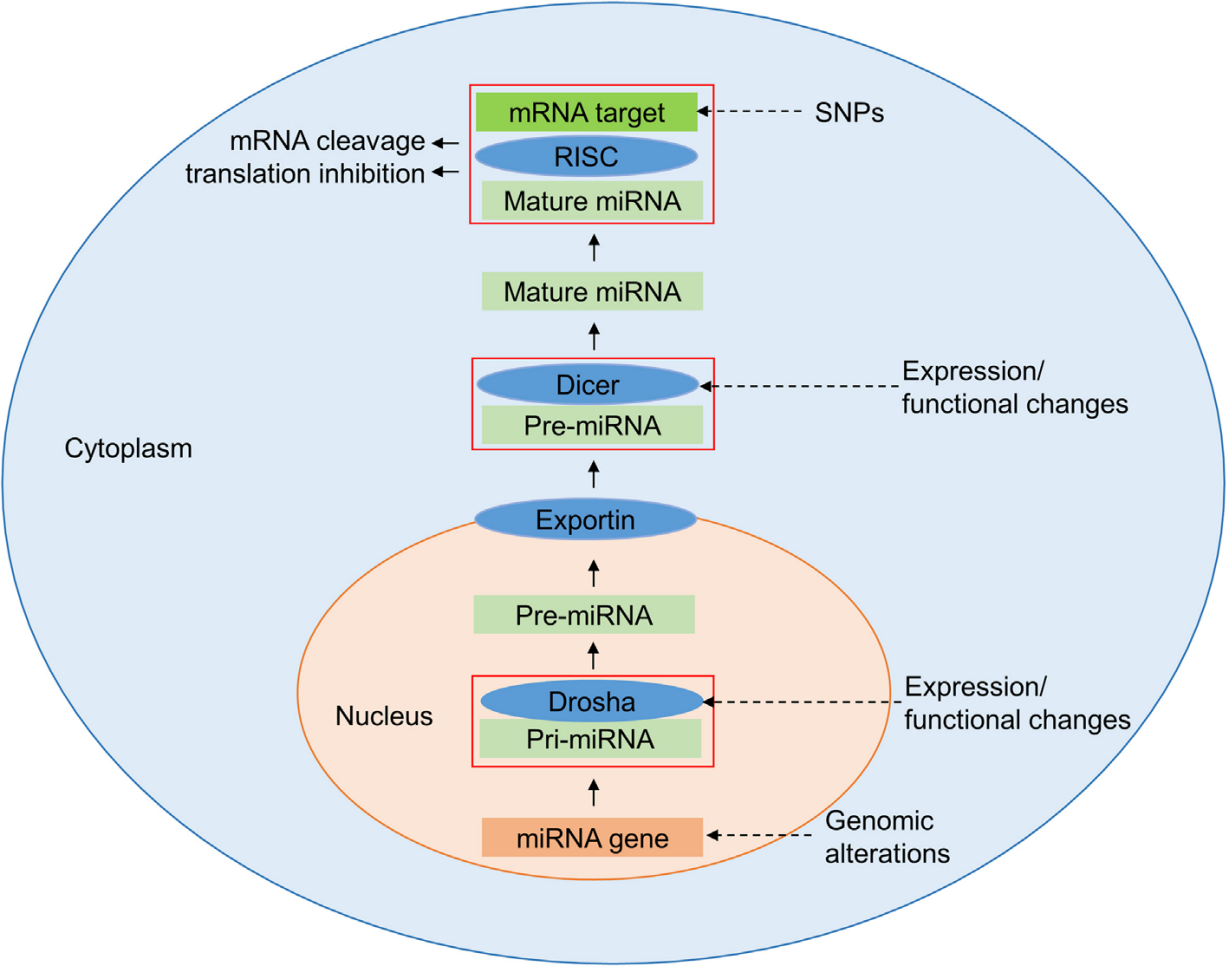
**Regulation of microRNAs (miRNAs)**. Expression of miRNAs can be altered at various stages of its biogenesis by genomic [single-nucleotide polymorphisms (SNPs) and mutations] and epigenetic alterations. Changes in the expression and function of Drosha and Dicer, part of the miRNA processing machinery, lead to the deregulation of mature miRNAs. The figure has been adapted from Ref. (14, 15).

**Figure 2:**
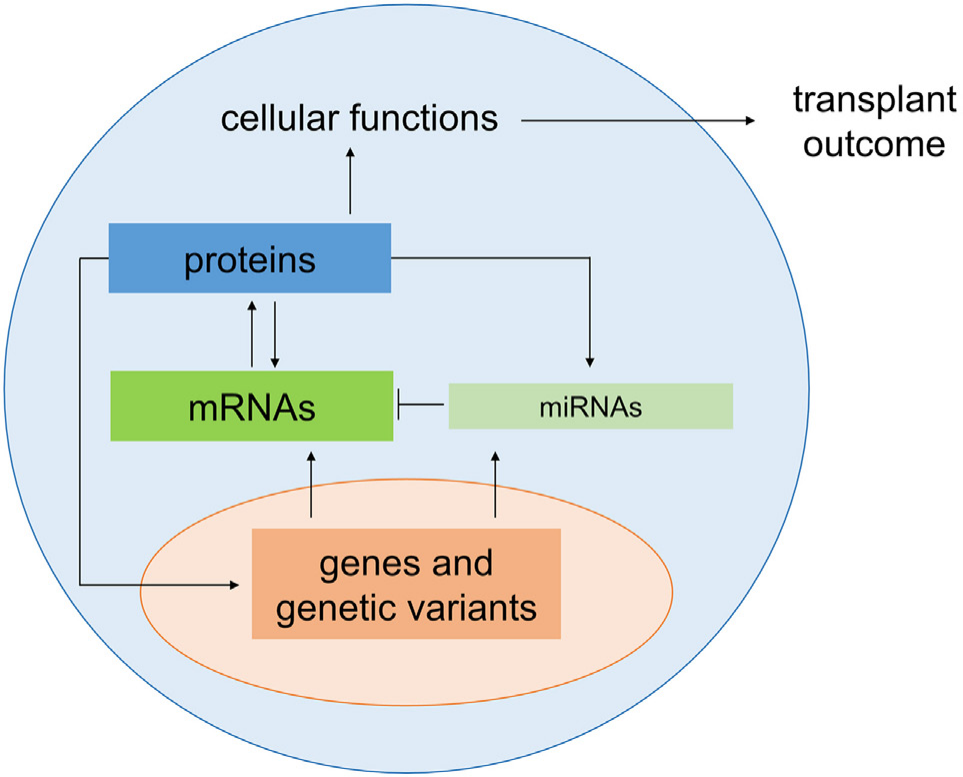
**Genetic regulation of transplant outcome**. Genetic variants in protein encoding genes and microRNAs (miRNAs) alter gene expression as well as protein and cellular functions which in turn contribute to the regulating the outcome of transplantation. The color code introduced here is used for genes, miRNAs, mRNAs, and proteins in Figures 3–5.

The most frequent genetic variations of the human genome are single-nucleotide polymorphisms (SNPs), which occur on average in 1 out of 300 bp throughout the genome (4–6). The majority of the SNPs arise in non-coding regions including intronic, intergenic, and untranslated regions (UTRs) (7). Those which are within genes, including genes affecting the immune response, may alter the expression of the gene or the structure of the encoded proteins (8). In microRNAs (miRNAs), SNPs can alter regulatory properties, but elucidation of the functions of these SNPs is not straight forward (9). Understanding the biogenesis of miRNAs is key to comprehending the impact of SNPs on these molecules (Figure 1). The miRNAs are a class of small endogenous non-coding RNAs of 21–25 nucleotides in length that originate as primary transcripts (pri-miRNAs) from miRNA genes. After transcription of pri-miRNAs by RNA polymerase II, they are processed by DROSHA, a RNA specific ribonuclease enzyme complex, producing short precursor-miRNAs (pre-miRNAs) of approximately 70 nucleotides length (10). The pre-miRNAs are then transported from the nucleus to the cytoplasm by exportin 5 (10). In the cytoplasm, they undergo further cleavage by an endonuclease enzyme (DICER), resulting in the generation of mature miRNA (11, 12). Accordingly, functionally relevant SNPs can be present in miRNA biogenesis-related genes, in specific miRNA-encoding genomic loci or in the seed match sequence of target mRNA 3′ UTRs. SNPs may lead to either an alteration in miRNA expression level, a decreased or increased miRNA-target interaction, or a new miRNA-target interaction (13). Atarod and Dickinson (14) described the driving gears of GvHD as miRNA’s regulating gene expression, chemokine and cytokine secretion, while their expression in turn is affected by SNPs in mRNA genes (Figure 2).

In this review, we will pinpoint SNP–mRNA–miRNA regulatory network alterations and their contribution to the risks associated with HSCT in specific examples, elucidating the consequences of the interaction between these three genetic elements. Moreover, we will summarize mRNA and miRNA profiling studies aiming to decipher genetic risks of HSCT.

## Examples of SNP–mRNA–miRNA Regulatory Networks Controlling Outcome of HSCT

### Forkheadbox Protein 3 (FOXP3) Polymorphisms, mRNA Expression, and FOXP3-Regulating miRNAs

Regulatory T cells (T_regs_) have been the focus of several HSCT studies due to their ability to suppress alloreactivity during GvHD (16). T_regs_, defined as CD4^+^CD25^+^ FOXP3^+^ T cells, are involved in the maintenance of immunological tolerance (17). They reduce the invasion of CD8^+^ effector T cells (T_eff_) in target tissue and ameliorate GvH tissue damage (18). Cuzzola and colleagues found an increased expression of *FOXP3* mRNA in patients who were responsive to anti-GvHD therapies (19). These results are in concordance with previous data reporting an inverse correlation between the amount of T_regs_ and progression of aGvHD (20). Other studies showed correlation between a lower incidence of aGvHD and improved survival in HSCT recipients with an increased number of donor T_regs_ (21, 22). Low numbers of T_regs_ have also been associated with higher cGvHD incidence (23). Similarly, the severity of aGvHD and extent of cGvHD in patients were found to be associated with T_reg_ numbers (24). Furthermore, inducing selective expansion of T_regs_ by the daily administration of low doses of interleukin (IL)-2 showed an improvement in clinical cGvHD symptoms in patients (25). Notably, not only CD4^+^ T_regs_ can mitigate GvHD but also CD8^+^FOXP3^+^ T_regs_ become induced during GvHD and can suppress the disease in mouse models (26, 27). CD8^+^ T_regs_ might have even advantageous over CD4^+^ T_regs_ since they have been reported not to abrogate graft-versus-leukemia (GvL) effects (28). The potency of CD8^+^ T_regs_ cells is further emphasized by their ability to prevent the rejection of heart allografts in rats (29).

**Figure 3:**
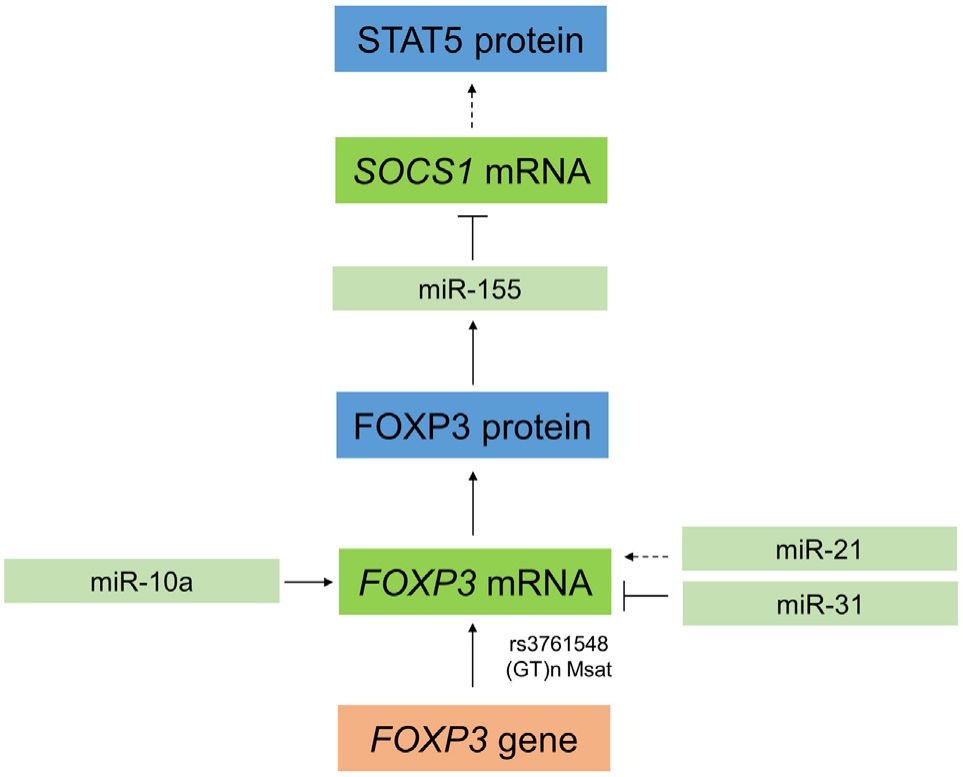
**Interaction between microRNAs and forkheadbox protein 3 (FOXP3) in regulatory T cells (T_regs_)**. In T_regs_, miR-10a stabilizes *FOXP3*, and FOXP3 can positively regulate expression of miR-155. This leads to a downregulation of the target *SOCS1* (⊤), which in turn results indirectly in increased expression of STAT5. *FOXP3* can also be regulated by miR-21, which indirectly positively regulates *FOXP3* in a process that is not yet completely understood. Moreover, *FOXP3* is downregulated by miR-31 by direct targeting of the 3′ untranslated region. To further complicate this regulatory network, single-nucleotide polymorphisms (SNPs) in *FOXP3* also affect its expression in T_regs_, such as the SNP rs3761548 or a GT(n) microsatellites (Msat).

Currently, 90 SNPs have been identified in the *FOXP3* gene region, and several have been identified as risk factors for a number of malignant and autoimmune diseases (30). An SNP (rs3761548) in the promoter region of *FOXP3* (Figure 3) resulting in an *A*/*C* base exchange causes loss of binding to the E47 and c-Myb factors and leads to defective transcription of the *FOXP3* gene (31). In patients undergoing HSCT, this SNP has been associated with a higher incidence of hepatic veno-occlusive disease and cytomegalovirus (CMV) infection but a lower treatment-related mortality, resulting in a difference in the overall survival of patients with the *CC* genotype (32). However, the authors found no difference in the incidence of GvHD, relapse, or blood stream infection to be associated with this polymorphism (32).

Posttransplant chimerism analysis in clinical HSCT largely uses polymorphisms of short tandem repeats of <10 nucleotides, or microsatellites (Msat). They have a higher degree of allelic polymorphism compared to SNPs, and therefore, a larger degree of information (33). An Msat studied in *FOXP3* is the (*GT*)n polymorphism in the promoter/enhancer region of the *FOXP3* gene (34). This polymorphism was shown to be associated with the development of auto- or alloimmune conditions, including type I diabetes, and graft rejection in renal transplant recipients (35). Moreover, this polymorphism has been associated with a lower incidence of grade III–IV GvHD in patients transplanted from donors carrying short alleles [≤(*GT*)15] (35). However, this polymorphism did not affect relapse, event free survival or overall survival in patients with aGvHD and cGvHD (35).

Recently, several miRNAs including miR-155 and miR-10a have been identified that impact T cell differentiation and function (36, 37). MiR-155 is required for T_reg_ development (38) and for maintaining T_reg_ homeostasis and survival by targeting *SOCS1* (39). MiR-155 is an important positive regulator of natural T_reg_ (nT_reg_) development and miR-155 gene transcription is driven by FOXP3 (Figure 3) (38). In mice, miR-155 is upregulated in mature T_regs_ (CD4^+^CD25^+^FOXP3^+^) relative to conventional T cells (CD4^+^CD25^−^FOXP3^−^) as well as in FOXP3^+^ double positive and single positive thymocytes (37, 40). MiR-155 knockout mice have reduced T_reg_ numbers in both the thymus and periphery, and miR-155-deficient T_regs_ have a reduced proliferative potential and impaired IL-2 signaling (39). In this context, miR-155 promotes T_reg_ survival and proliferation in the thymus and periphery by enhancing their sensitivity to IL-2 (39). As shown in Figure 3, miR-155 achieves this by targeting and downregulating *SOCS1*, an inhibitor of IL-2 signaling, thus increasing levels of activated STAT5 and enhancing IL-2 signaling (40). MiR-10a is functionally linked to stabilization of FOXP3 in T_regs_ (41) (Figure 3) and interestingly, although miR-10a has not been specifically investigated in relation to HSCT, an inverse correlation between miR-10a and susceptibility to autoimmune disease has been identified (41). With regard to miR-10a, it is uniquely expressed in T_regs_, but not other T cells, where it is crucial for long-term maintenance of their stability and function (41). *FOXP3* itself can be regulated by other miRNAs, including miR-21 and miR-31, which positively and negatively regulate *FOXP3*, respectively, thus having opposing effects on its expression (36) (Figure 3). MiR-31 can directly target *FOXP3* by binding to a specific recognition site in the 3′ UTR region, while miR-21 regulation is believed to be indirect as no potential target sequence in *FOXP3* was identified (36). The specific function of miR-21 and miR-31 in T_regs_ in the setting of HSCT is still to be explored.

### The miR-155 and miR-146a Regulatory Network

**Figure 4:**
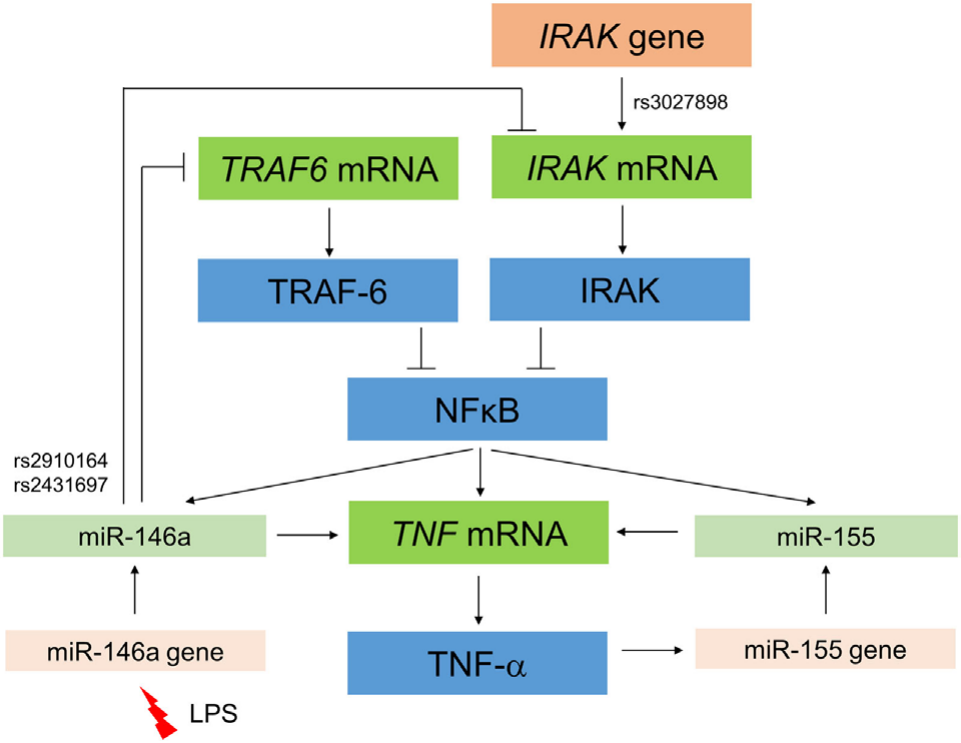
**Interaction between miR-146 and miR-155, their effects on the nuclear factor (NF)-kB pathway, and the expression of IRAK1 and tumor necrosis factor (TNF)-α**. Activation of the NF-kB pathway represents a hallmark of the pathophysiology of GvHD. NF-kB activation induces expression of miR-146a and in turn, miR-146a inhibits these pathways through targeting key adapter proteins, IRAK1(⊥) and TRAF6 (⊥). The presence of single-nucleotide polymorphisms in coding regions of these genes, such as rs3027898 in *IRAK1*, further influences expression within the network. MiR-146a expression can also be stimulated by lipopolysaccharide (LPS) release during GvHD conditioning. The miR-146a and miR-155 mediate an increase in TNF-α, which in turn can positively regulate miR-155 in a feedback loop.

Notably, miR-155 is involved in a larger regulatory network that affects the outcome of HSCT (Figure 4). Although miR-155 promotes the development of T_regs_ as explained above, it may also have pro-inflammatory functions. MiR-155 and miR-146a were found to be upregulated in the skin of rats suffering from aGVHD (42). Atarod and colleagues showed that low expression levels of both miR-155 and miR-146a were associated with higher incidence of aGVHD at day 28 post-allo-HSCT in patients and that both regulate expression of the transcription factor SPI1 (43). Pontoppidan and colleagues showed that miR-155 was increased in patient plasma at the time of maximal toxicity of preconditioning at day 7 posttransplantation and remained increased until day 21. This was inversely mirrored by miR-146a, which was significantly reduced from day 7 to day 21 after transplantation (44). Together, this suggests that miR-155 and miR-146a play opposite roles having pro-inflammatory and anti-inflammatory properties, respectively, and thus play a role in regulating the systemic inflammatory response during maximum toxicity of preconditioning in HSCT patients (44). Relevantly, miR-155 expression was upregulated in donor T cells in mice during aGvHD and mice receiving miR-155-deficient splenocytes developed less severe aGvHD and had increased survival rates compared to mice receiving wild type splenocytes (45). Specific targeting of miR-155 using antagomirs effectively mitigated aGvHD in mice and increased survival rates (45). MiR-155-deficiency in the dendritic cell (DC) compartment also protected mice from aGvHD since miR-155 appears to promote the migration of DC toward sites of tissue damage (46). MiR-155 expression was increased in mouse T cells as well as in intestinal patient biopsies during aGvHD (45). Expression of miR-155 can be stimulated by tumor necrosis factor (TNF) α (47), and similarly, miR-155 can promote TNF-α production in a positive feedback loop (48), thus exacerbating the inflammatory cascade (Figure 4). Altogether, these data indicate a role for miR-155 in the modulation of aGvHD.

MiR-146a is distinctly increased in response to lipopolysaccharide (LPS), a component of the cell wall of gram-negative bacteria. LPS is released in response to GvHD conditioning regimens (Figure 4) and acts as a potent enhancer of cytokine secretion (49). Using a genetically engineered mouse model, it was demonstrated that a deletion of miR-146a results in several immune pathologies (50). Specifically, lack of miR-146a expression increased responsiveness of macrophages to LPS and exacerbated the inflammatory response in LPS-challenged mice. TNF receptor-associated factor 6 (*Traf6*) and IL-1 receptor-associated kinase 1 (*Irak1*) genes have also been identified as targets of miR-146a (Figure 4), contributing to the phenotype of miR-146a-deficient mice (50). Both TRAF6 and IRAK1 act as adapter proteins in the nuclear factor (NF)-κB activation pathway and in addition to innate immune cells, miR-146a has also been shown to target these genes in T cells resulting in their downregulation (Figure 4). T cells that are lacking miR-146a are hyperactive in both acute antigenic responses and chronic inflammatory autoimmune responses (51). However, the presence of SNPs in these genes that may affect miR-146a binding, such as rs3027898 in the *IRAK1* 3′UTR may further complicate this already complex network of interactions. Furthermore, activation of NF-κB has been described to upregulate miR-146a expression, which in turn downregulated NF-κB *via TRAF6* and *IRAK1* repression in a negative feedback loop (52, 53) (Figure 4). More recently, miR-146a regulation of *TRAF6* and *IRAK1* was specifically associated with aGvHD, since upregulation of miR-146a expression was observed in T cells of mice developing aGvHD compared to untreated mice (52). When transplanted with miR-146a-deficient T cells, recipient mice developed GvHD of increased severity resulting in reduced survival, and they also had elevated TNF-α serum levels (52). The protein levels of TRAF6 were upregulated in miR-146a-deficient T cells following alloantigen stimulation, and this translated into increased NF-κB activity. Conversely, miR-146a overexpression reduced aGvHD severity. In an autoimmune setting, miR-146a expression can be induced by TNF-α (47), which in turn is stimulated by NF-κB, thus further confounding this complex regulatory network (Figure 4). Interestingly, another member of the miR-146 family, i.e., miR-146b, is highly expressed in CD4^+^CD25^+^FOXP3^+^ thymic-derived T_regs_ and has been reported to promote survival, proliferation, and suppressor function of these cells by targeting *TRAF6* and subsequently increasing NF-κB activity (54).

Single-nucleotide polymorphisms within miRNA coding regions as well as those within their target mRNA seed regions can directly influence miRNA–mRNA interactions. Indeed, with regard to miR-146a, two SNPs rs2431697 and rs2910164 have been reported that cause single base changes and altered expression of the mature microRNA (Figure 4). The SNP rs2910164 specifically results in a change from a G:U pair to a C:U mismatch in the stem structure of the miR-146a precursor. This results in processing variation and lower expression of the mature miRNA, which has been associated with the development of a range of cancers (55). Stickel and colleagues reported that the minor *CC* genotype caused a decrease in miR-146a production (52). The same team also provided evidence that miR-146a acts as an important negative regulator in murine and human GVHD, consistent with an anti-inflammatory role for miR-146a, and suggested the exogenous increase of miR-146a as a potential novel strategy for therapeutic intervention in this disease (52). Further interactions that have been described between miRNAs and the induction of GvHD have been recently reviewed by Atarod and Dickinson (14).

### MICA Polymorphisms, mRNA Expression, and MICA-Regulating miRNAs

**Figure 5:**
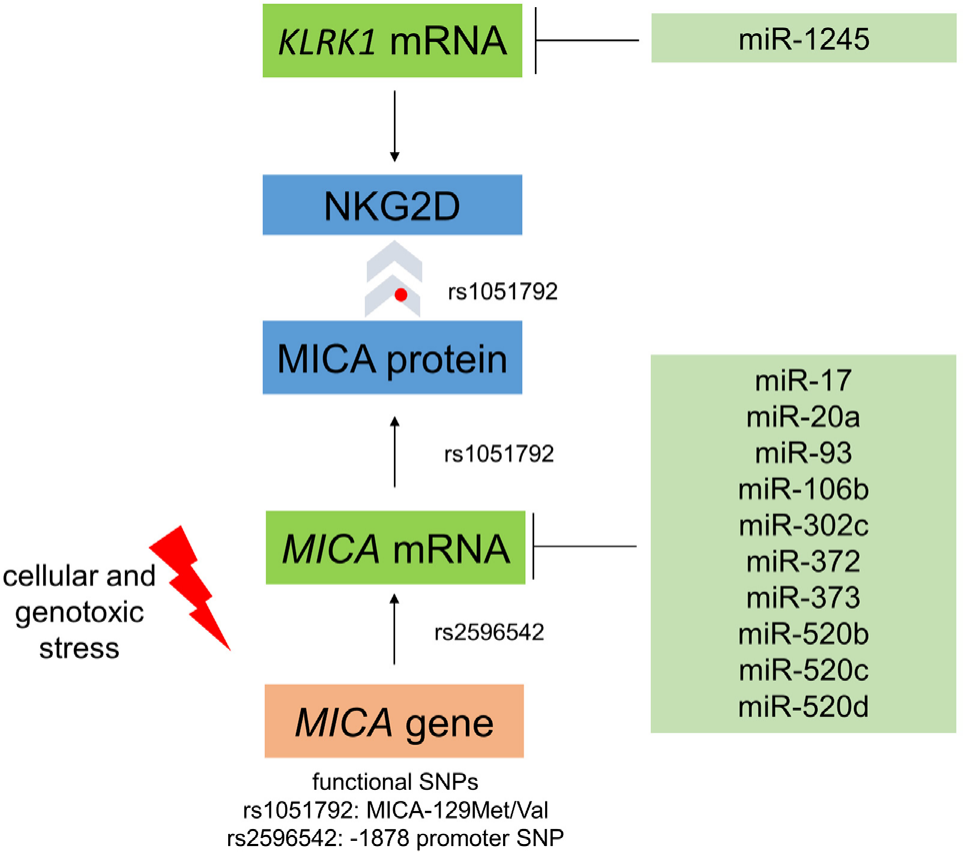
**Regulation of MICA expression and interaction with NKG2D**. The single-nucleotide polymorphism (SNP) rs1051792 results in a valine to a methionine exchange at position 129 of MICA and distinguishes MICA variants into those binding the receptor NKG2D with high (MICA-129Met) or low (MICA-129Val) avidity. This polymorphism also affects the cell surface expression of MICA protein. The SNP rs2596542 in the promoter of *MICA* affects mRNA expression. Moreover, several microRNAs target *MICA* and downregulate its expression. Moreover, cellular and genotoxic stress induces the expression of MICA. The MICA receptor NKG2D is encoded by the *KLRK1* gene and can be targeted by miR-1245.

The major histocompatibility complex (MHC) class I chain-related molecule A (MICA) is a highly polymorphic ligand for the activating natural killer (NK) cell receptor NKG2D (Figure 5). An SNP within this gene, rs1051792, which leads to an amino acid exchange from valine to methionine at position 129 (56), was investigated for its association with the outcome of HSCT. We found that the MICA-129Met variant was associated with an increased overall survival and a reduced risk to die from aGvHD, despite homozygous carriers of the *MICA-129Val* allele having an increased risk of developing aGvHD (57). The NKG2D pathway was expected to be directly related to the outcome of HSCT, since it is an activating receptor on NK cells (58) and a costimulatory receptor on CD8^+^ T cells (59). On the functional level, we found the MICA-129Met isoform triggered more cytotoxicity and interferon (IFN)-γ release by NK cells and it activated alloreactive cytotoxic T cells faster. This variant also induced more rapid and severe downregulation of NKG2D on NK and cytotoxic T cells (57). Normally, most cell types do not express MICA, but it becomes induced by cellular and genotoxic stress, including virus infection and malignant transformation. Therefore, it renders stressed cells susceptible to killing by NK cells and allows them, despite being non-professional antigen presenting cells (APCs), to directly activate cytotoxic T cells specific for antigens presented by these cells. Notably, MICA expression was found to be increased in GvHD-affected tissue samples from patients (60). The MICA-129Met variant can therefore initially confer a higher risk of aGvHD due to a faster activation of alloreactive cytotoxic T cells (57). However, in the longer perspective, the strong-counter regulation of NKG2D by this variant appears to be associated with a decreased risk of cGvHD and an increased risk of relapse due to lesser GvL effects by cytotoxic T cells and NK cells (61).

Interestingly, the biological effects of the MICA-129 variants were strongly influenced by MICA expression intensity (57). The MICA-129Met variant triggered increased NKG2D signals at low expression intensities, whereas the MICA-129Val variant elicited more NKG2D-mediated effects at high expression intensities. At high expression intensity, the functional effects of the MICA-129Met variant were impaired due to a rapid downregulation of NKG2D (57). Thus, MICA expression intensity could change the biological effect of this SNP, giving an interesting example of the complex functional interactions between SNPs and gene expression (Figure 5). Moreover, the SNP might interact with other SNPs in the NKG2D signaling pathway (62) including *KLRK1*, encoding NKG2D. Polymorphisms in the *KLRK1* locus have been described also to affect the outcome of HSCT (63).

Notably, MICA expression intensities can vary for certain *MICA* alleles (64). The SNP at −1878 (rs2596542) in the promoter region of the *MICA* gene was described to affect the transcriptional activity (65). A polymorphic microsatellite in exon 5 encoding the transmembrane region of MICA modifies its plasma membrane expression (66). We have recently shown that the MICA-129Met/Val dimorphism also affects plasma membrane expression. Increased levels of the MICA-129Met variant were retained intracellularly and if expressed at the cell surface, the MICA-129Met variant was more prone to shedding than the MICA-129Val isoform (67).

Matching of donor and recipient for *MICA* alleles (68–71) and specifically for the *MICA-129*, polymorphism (72) has been found to be beneficial in HSCT, although not in all studies (73, 74). The effect of *MICA* matching appears hardly explainable solely by the avoidance of potential miHAg and further points toward an important biological function of MICA after HSCT.

Several stress pathways regulate the transcription of the *MICA* gene (75), and several miRNAs have been implicated in controlling MICA expression *via* posttranscriptional mechanisms (Figure 5). Stern-Ginossar and colleagues described that the expression of *MICA* was decreased by miR-17, miR-20a, miR-93, miR-106b, miR-372, miR-373, and miR-520d (76). Effects of miR-17 (77), miR-20a (77–80), miR-93 (77, 80–82), and miR-106b (80, 81) on *MICA* expression have also been confirmed in subsequent studies. Moreover, the IFN-γ-induced miR-520b can lead to a reduction in MICA plasma membrane expression intensity (83). Interestingly, miR-520d acts on both the *MICA* 3′-UTR and the promoter region to decrease *MICA* transcript levels (83). MiR-302c and miR-520c are two further miRNAs that can target *MICA* (84). Human CMV can also target MICA by US18 and US20, which promote the degradation of MICA in lysosomes (85). The regulation of NKG2D ligands by miRNAs has been recently reviewed in more detail (86, 87). Notably, also the expression of NKG2D has been found to be attenuated by miRNAs, specifically, miR-1245 (88).

## miRNA and mRNA Expression Profiling in HSCT

A number of large-scale gene expression profiling studies have been performed in both patients and animal models to identify genes regulated during HSCT. In animals, both acute and chronic GvHD models were investigated. Studies performed on human samples mostly used blood or in cases of cGvHD, conjunctiva from patients. The advantage of using animal models for gene expression is the broad availability of specific target tissues of GvHD, such as liver and skin (89). A selection of the most relevant gene expression profiling studies is listed in Table 1. MiRNAs have also been studied in relation to HSCT, and there is increasing evidence to show that miRNAs are present in plasma, serum, saliva, urine, and other body fluids in a remarkably stable form that is protected from endogenous RNase activity (90). Circulating miRNAs have the potential to serve as novel and non-invasive biomarkers for various diseases such as cancer, cardiovascular disease, and organ transplant rejection and infection (91).

**Table 1:** **Summary of large-scale mRNA expression profiling studies during GvHD**.

Species	Tissue	Disease	Upregulated	Downregulated	Technique	Reference
Human	Peripheral blood mononuclear cells (PBMCs)	Acute graft-versus-host disease (aGvHD)	*CXCL8, GOS2, ANXA3, NR4A2*	*CDKN1C*	qRT-PCR	(92)
Human	PBMC	aGvHD	*PCDHB5, IL22RA2, PDCDILG2, IL2, PKD1*	*PCDHB16, IL27, IGHD, CCL1, CF1*	Microarray	(93)
Human	CD4^+^ and CD8^+^ T cells	Chronic graft-versus-host disease (cGvHD)	*EP300, FURIN, FNBP3, SMAD3, TGFBI, TGIF*	*PRF1*	qRT-PCR	(94)
Human	PBMC	aGvHD	*TNFSF10/TRAIL, IL1RN, IFI27, GZMB, CCR5*	*CLK1, TNFAIP3*, and *BTG1*	Microarray	(95)
Human	PBMC	cGvHD	*IL21R IL18, CD28, IL17A, IL6R, PI3K*	*IFNG, CCl1, IRAK3, IL12B, SOC2*	Microarray	(96)
Human	Conjunctiva	cGvHD-DE	*CXCL9, CXCL10, CXCL11, CXCR3*		qRT-PCR	(97)
Human	Conjunctiva	GvHD-DE	*IL6, IL9, IL10, CCL24, CCL28, CCL2*	*EGFR*	qRT-PCR	(98)
Mouse	Ear skin day 7	aGvHD	*Saa3, Cxcl9, Cxcl10, Ccl5, Ubd*	*Trp1, Col1a1*	Microarray	(99)
	Ear skin day 40		*Ccl5, Saa3, Il1beta, Cxcl9*	*Ces3*		
Mouse	Liver day 7	aGvHD	*Vcam1, Cxcl9, Gbp2, Tgtp, Tap1*	*Lck, Ltf*	Microarray	(100)
	Liver day 35		*Ccl5, Icam1*	*Lck, Ltf*		
Mouse	Liver versus kidney	aGvHD	*Cxcl9, Cxcl10, Vcam1, Stat1, Icam1*		Microarray	(101)
Mouse	Scl^a^ skin	GvHD	*CXCL5, CXCL11, CXCL9, CXCL10, IL6, TGF-BR1*	*Cdh5, Cdh13*	Microarray	(102)
Rat	Skin explant model	aGvHD	*RT1-CE10 Lst1, Ubd, Aif1, RT-DMb*	*Ly6g6e, Bat5*	Microarray	(103)

*The listed mRNA profiling studies were carried out with human or rodent tissues obtained during both aGvHD and cGvHD. The genes regulated during GvHD with the highest fold changes and significant P-values are indicated*.

*^a^Scl—sclerodermatous skin model of GvHD*.

### miRNAs Targeting Immune-Related Genes and Their Application on Prediction of HSCT Outcome

In a recent clinical study, a micro-RNA-based model was developed to predict the probability of aGvHD comprising miR-423, miR-199a-3p, miR-93 and miR-377. Elevated levels of these miRNAs were detected in plasma before the onset of aGvHD (average 16 days before diagnosis), and their expression was associated with the severity of aGvHD as well as with a lower overall survival (91). In another profiling study of 48 miRNAs in the plasma of aGvHD patients, miR-586 expression was decreased upon the occurrence of aGvHD (104).

Interestingly, a recent publication by Wu and colleagues (105), which has been reviewed by Serody (106), has been shown that the miR-17–92 cluster, which is conserved among vertebrates, is important for the development of aGvHD. The miR-17–92 cluster is critical for the proliferation, survival and function of Th_1_ and Th_17_ effector T cells and inhibits Th_2_ and T_reg_ differentiation (107). It has now also been shown that this cluster of miRNAs promotes the migration of CD8^+^ T cells to GvHD target organs and confers GvL effects (105). Donor T cells lacking the miR-17–92 complex gave rise to diminished GvHD in a mouse model of aGvHD. Blocking of miR-17 and miR-19b, which are included in the miR-17–92 complex, by systemic administration of antagomiRs (locked nucleic acid-modified oligonucleotides) significantly reduced aGvHD severity (105).

To better understand the regulation of neovascularization during GvHD, Leonhardt and colleagues focused on the role of miR-100 and showed that in intestinal tissue biopsies from patients undergoing allo-HSCT, miR-100 was downregulated when aGvHD evolved suggesting that this miRNA has a role as a negative regulator of aGvHD (108). MiR-100 was also downregulated in the inflamed intestinal tissue of mice developing aGvHD. In the mouse model, functional inactivation of miR-100 with antagomiRs enhanced aGvHD severity, indicating a protective role for miR-100 by blocking inflammatory neovascularization during aGvHD (108).

Other studies focusing on miR-34 showed that the miR-34 family mimics p53 effects by inducing cell cycle arrest and apoptosis in response to DNA damage (109). In the case of Fanconi anemia (FA), an inherited disorder characterized by developmental defects, genomic instability and progressive bone marrow failure (110), miR-34a expression in patient gut biopsies after HSCT was significantly higher in aGvHD grades II–IV compared to grade 0–I or with non-transplanted FA patient gut biopsies (111).

### Association between Polymorphisms and Gene Expression Levels in Cytokine and Chemokine-Coding Genes with the Outcome of HSCT

#### Cytokine Gene Expression Variation and Its Impact on the Outcome of HSCT

Cytokines, such as INF-γ, IL-2, and TNF-α produced by Th1 cells, contribute to the induction phase of aGvHD (112). A number of polymorphisms in genes encoding IL-10, TNF-α, and IL-6 have been linked to an increased risk of GvHD. IL-2 has a role as a T cell growth factor and treatment, and prophylaxis of aGvHD involves frequently inhibition of IL-2 production by cyclosporine A (113). Moreover, in both animal and human studies, administration of monoclonal antibodies against the IL-2 receptor after HSCT have prevented aGvHD occurrence (114, 115). On the other hand, emerging data show that IL-2 is also necessary for the generation and the maintenance of CD4^+^CD25^+^Foxp3^+^ T_regs_, so that inhibiting IL-2 may have a negative effect on the development of long-term tolerance after allo-HSCT (116, 117). Another cytokine of critical importance during aGvHD is IFN-γ, a pro-inflammatory cytokine, produced by several cell types such as activated T cells, NK, and NKT cells. IFN-γ and IL-2 both play a role in T cell proliferation, stimulation of cytotoxic T lymphocyte (CTL) and NK cell responses and production of IL-1 and TNF-α (118). A number of studies have reported a correlation between the expression of IFN-γ and severity of aGvHD (119–121). IFN-γ production occurs early in the cytokine cascade of GvHD. Acute GvHD is augmented by IFN-γ, which leads to the maturation of DC and stimulation of macrophages to generate cytokines and NO (118).

*IFNG* (IFN-γ) mRNA expression in the conjunctiva of patients was associated with dry-eye cGvHD (98) and in CD8^+^ T cells with cGvHD (122). Ichiba and colleagues studied the regulation of 7,329 genes in the hepatic tissue of mice on days 7 and 35, after allogeneic and syngeneic bone marrow transplantation (100). On day 7, 456 genes and on day 35, 554 genes were regulated. Interestingly, *Ifng* mRNA expression was not upregulated during hepatic GvHD on day 7 and no expression of *Ifng* was observed in the liver on day 35. However, the expression of many genes that are inducible by IFN-γ, such as interferon regulatory factor-1 (*Irf1*) and *Irf7* were increased (100). Both *IFNG* and *IL2* mRNA were increased in the peripheral blood mononuclear cells (PBMCs) of GvHD patients that received a donor lymphocyte infusion for the treatment of relapsed leukemia after allogeneic HSCT and *IL2* mRNA expression correlated with the progression of GvHD (121). In another study, genes that contribute to control of inflammation, such as the IL-1 decoy receptor *IL1R2*, as well as pro-fibrotic genes have been found to be overexpressed in PBMCs of patients with cGvHD (123).

Gene expression of *TNF*, encoding TNF-α, a critical pro-inflammatory cytokine, was also elevated during aGvHD in PBMCs of patients (93). TNF-α is one of the most important factors involved in the pathogenesis of aGvHD, and it is important at various stages during the progression of the disease. The importance of this molecule in aGvHD was firstly described in a mouse model (124). Since then, several studies have shown that the neutralization of TNF-α can reduce symptoms of aGvHD (125). Tumor necrosis factor superfamily, member 10 (*TNFSF10*) mRNA expression was also elevated during aGvHD in human PBMCs (95).

Other cytokines regulated in GvHD include IL-15. In a murine aGvHD model, donor IL-15 was crucial for the development for aGvHD (126). Investigations on the role of IL-15 suggested that IL-15 could induce aGVHD by activating T cells and NK cells (127). In conjunctiva of patients with GvHD, *IL15* mRNA was significantly increased (98). *IL27* mRNA was strongly downregulated during aGvHD in PBMCs from patients (93). Previous reports indicate that IL-27 exhibits a pro-inflammatory response, is involved in activating Th1 cells, and enhances the immunological response to tumor cells (128). IL-35 is an inhibitory cytokine secreted by T_regs_. The exact role of IL-35 in aGvHD is not known, although overexpression of IL-35 during murine aGvHD reduced its severity by suppressing activation of effector CD4^+^ T cells and expansion of CD4^+^Foxp3^+^ T_regs_ in target organs of aGvHD, while preserving a GvL effect (129). IL-35 could be a potential therapeutic target for prevention of aGvHD (129, 130).

#### SNPs in Cytokine Coding Genes and Association with HSCT Outcome

Given the dysregulation of many cytokines during acute and chronic GvHD, it is not surprising that SNPs in these genes have been associated with the outcome of HSCT. SNP association studies in HSCT have been recently reviewed by Dickinson and Norden (8). Since the original work by Middleton and colleagues (131), large cohort candidate gene association studies have been reported on SNPs in more than 20 genes that code for cytokines and other molecules involved in the biology of HSCT (132–135). Moreover, SNPs originally identified in *NOD2* for their association with Crohn’s disease have since been associated with HSCT outcomes (136, 137). Individuals carrying just one variant of rs2066844 (SNP8), rs2066845 (SNP12), or rs41450053 (SNP13) have a twofold to fourfold increased risk of developing Crohn’s disease, which increases to approximately 20-fold in individuals who are homozygotes or compound heterozygotes (138, 139). NOD2 is mainly involved in defense against infection; it recognizes pathogen-associated patterns and induces a cytokine response, and is itself regulated by pro-inflammatory cytokines (140).

Goussetis and colleagues retrospectively analyzed specific polymorphisms in genes for IL-10, IL-6, TNF-α, and IFN-γ in a pediatric cohort of 57 HLA-identical sibling myeloablative transplants and found a significant association between the *IL10* promoter haplotype polymorphisms at positions −1082, −819, and −592 with the occurrence of severe aGVHD (grades III–IV). Recipients with the haplotype *GCC* had a decreased risk of severe aGVHD in comparison with patients with other *IL10* haplotypes (141). Chien and colleagues identified two SNPs in *IL10*, such as rs1800871 and rs1800872, which were associated with a 30% decrease of the risk for grade III–IV aGVHD (139). Moreover, the donor allele *C* for rs1800795 in *IL6* was associated with a 20–50% increase in the risk for grade II–IV aGVHD, and the *IL2* polymorphism rs2069762 in the donor genotype was associated with a 1.3-fold increase in risk of grade III–IV aGVHD (139).

#### Chemokine Gene Expression Variation and Its Impact on the Outcome of HSCT

Many genes encoding chemokines are regulated during GvHD. CXCR3 is an important chemokine receptor involved in lymphocyte recruitment and is expressed on T cells. CXCL9, CXCL10, and CXCL11, the ligands for CXCR3, are induced by the Th_1_ cytokines IFN-γ and TNF-α (142). CXCL9 is expressed by effector CD4^+^ Th1 cells and CD8^+^ CTL and has been shown to affect the migration of T_eff_ to inflamed tissue during progression of GvHD (142). *CXCL10* and *CXCL11* mRNA expression were increased in patient skin biopsies with aGvHD (grades II–III) when compared to patients without or grade I GvHD (143). The mRNA expression of *CXCL9* and *CXCL10*, along with their receptor *CXCR3*, was increased in cGvHD in conjunctival biopsies of 10 patients when compared to 10 healthy controls (97). Elevated mRNA expression of the CXCR3 ligands, *CXCL*9 and *CXCL10* in target organs of GvHD, shows that *CXCR3* could have a role in GvHD (144). *CXCL8*, encoding IL-8, was upregulated in PBMC from patients who developed aGVHD (92). Moreover, *Cxcl9* (101) and *Cxcl10* were also elevated during murine aGvHD (100). Interestingly, the use of CXCR3-transfected T_regs_, as a novel therapeutic strategy, resulted in decreased severity of GvHD due to attraction of T_regs_ to the target tissues of GvHD (145).

Other chemokines involved in the stimulation and activation of T cells in lymphoid tissue (*Cxcl1, Cxcl2, Cxcl9*, and *Cxcl20, Ccl2, Ccl5, Ccl6, Ccl7, Ccl8, Ccl9, Ccl11*, and *Ccl29*) and chemokine receptors (*Ccr1* and *Ccr5*) were elevated in the skin of mice during acute GvHD (99). The chemokines CCL2, CCL3, CCL4, and CCL5 are involved in the migration of donor cells to the target organs during GvHD (146). *CCR5* mRNA was also increased during in aGvHD human PBMCs (95) and in murine skin during GvHD (102). In addition, *Ccl2, Ccl5, Ccl17, Cxcl9 (Mig), Cxcl10 (IP-10)*, and *Cxcl11 (1-TAC)* mRNAs were also significantly regulated in mouse skin during GvHD (102). In conjunctival biopsies of patients with GvHD, the gene expression of *CCL24, CCL18*, and *CCL2* was highly increased (98). Another chemokine mRNA, *Ccl5* (RANTES), was elevated in the skin of mice during aGvHD (99) and profoundly upregulated during hepatic aGvHD (100).

### Differential Expression of Genes Involved in Antigen Processing and Presentation during GvHD

The role of MHC molecules is of critical importance to the development of GvHD. Both class I (HLA-A, B, and C in human) and class II (HLA-DR, DQ, and DP in human) determine not only the histocompatibility but are also responsible for controlling T cell recognition (147). Expression of class II HLA molecules by professional APCs, mainly in the gastrointestinal tract epithelium and skin, allows CD4^+^ T cells to recognize foreign antigens, possibly contributing to the specific organ sites of aGvHD (148). During the afferent phase of the pathogenesis of aGvHD, the release of cytokines such as IFN-γ leads to an increased expression of MHC molecules. On day 7 of hepatic aGvHD in mice, MHC class II genes, including *I-A*α, *I-A*β, *I-E*α, and *I-E*β, were overexpressed and remained upregulated at day 35 of aGvHD. On the other hand, the expression of the MHC class I genes was not regulated in this study. However, the genes that encode alternative proteasome subunits and that alternate peptide production associated with MHC class I molecules proteasome subunit beta 9 (*Psbm9*) and *Psbm8* (also known as lower molecular mass peptides LMP2 and LMP7) were increased in mouse liver during aGvHD (100). Moreover, *Tap1* and *Tap2* mRNAs were increased in mouse skin (99) as well as in liver during aGvHD (100). TAP1 and TAP2 are transporters associated with antigen processing 1 and 2, responsible for translocating peptides into the endoplasmic reticulum before loading on MHC class I molecules. In a rat skin explant model, an increase in expression of *Tap1* as well as *Psbm8* and *Ubd* mRNA during graft-versus-host reaction was observed (103). UBD, also known as FAT10, is involved in the proteasomal degradation of cytosolic proteins by providing a ubiquitin-independent signal (149). The differential expression of the MHC I and II genes in addition to genes involved in antigen processing that have been observed during GvHD is in agreement with the important role of MHC genes for HSCT outcomes.

### Involvement of the miHAg in Immune Responses during HSCT

Mismatches of polymorphic peptides between donor and recipients cause miHag that can also elicit an alloimmune response (150). The extent of the desired GvL versus the unwanted GvHD responses is dependent on the expression profiles of these genes. In humans, miHag are mostly restricted by HLA class I molecules. Previously, mismatches for HA-1, HA-2, and HA-5 between donor and recipient have been described to be associated with an increased risk of GvHD (151). In a gene expression profiling study to identify the risk of GvHD and relapse posttransplant, the mRNA expression of miHag was assessed in 311 HLA-matched siblings from a single center (152). The *HA-8* gene was expressed in almost all tissues, whereas *ACC-1* gene had a restricted profile. Nonetheless, both HA-8 and ACC-1 miHag mismatches were found to be associated with occurrence of cGvHD (152).

Notably, whole exome sequencing studies have been performed recently to estimate the alloreactive potential between donors and recipients in HSCT. It has been found that non-synonymous and non-conservative SNPs were twice as frequent in HLA-matched unrelated compared to related donor–recipient pairs (153). The information on SNPs between donor and recipient can be used to predict candidate miHags by algorithms taking peptide binding to HLA class I molecules and the tissue distribution of the respective proteins into account (154). Modeling of T cell responses to these miHags potentially can help to identify more favorable donors or to adapt the immunosuppressive treatment after HSCT (155, 156).

### Gene Expression Patterns in T Cells Associated with the Outcome of HSCT

Baron and colleagues compared the gene expression profiles of 50 allo-HSCT donors in CD4^+^ and CD8^+^ T cells to identify donors that are stronger allo-responders and could elicit a stronger GvHD response than others could. They suggested genes that regulate the transforming growth factor-β signaling and cell proliferation, in donor T cells, as the dangerous donor trait responsible for the occurrence of cGvHD in the corresponding recipients (94). Low levels of *SMAD3* mRNA, which encodes a transcription factor that is activated in response to TFG-β in CD4^+^ T cells, was associated with the absence of GvHD, while high levels of *SMAD3* were necessary but not sufficient for GvHD occurrence (94).

CD8^+^ T cells are important effectors in aGvHD (157), and perforin is a cytotoxic effector protease produced by CTL and NK cells. High expression of perforin 1 (*PRF1*) mRNA in CD8^+^ T cells was found to be associated with the incidence of GVHD in patients (94). In the skin of mice during aGvHD, granzyme B (*Gzmb*) was significantly elevated, in addition to the downstream effector caspase 7 (*Casp7*) (99). Other genes upregulated included the pro-apoptotic members of the BCL2 family, BCL2-antagonist/killer 1 (*Bak1*), BLC2-like 11 (*Bcl2l11*), and BCL2-associated X protein (*Bax*) (99). Thus, expression profiling can indicate ongoing pathophysiological processes contributing to GvHD, such as cellular cytotoxicity.

Notably, Sadeghi and colleagues observed an increase in gene expression of the adhesion molecules intracellular adhesion molecule 1 (*Icam1*) and vascular cell adhesion molecule 1 (*Vcam1*) during murine hepatic aGvHD. Increased expression of *Vcam1* mRNA was also observed in the liver and kidney compared to the muscle during murine aGvHD, whereas *Icam1* mRNA was upregulated only in the liver (101). Both adhesion molecules are expressed on endothelial cells and are critical for the migration of leukocytes to tissues during inflammation (158). Furthermore, *Icam1* and *Vcam1* genes were also upregulated in mouse skin during aGvHD, along with other adhesion molecules *Cd18* or integrin subunit beta 2 (*Itgb2*), *Ly69* or integrin beta 7 (*Itgb7*), and *Psgl1* or selectin platelet ligand (*Selplg*) (99).

The expression of costimulatory molecules that have a role in regulating T cell activation, differentiation, and proliferation has been studied to determine their role in GvHD. CD28 and CD28/cytotoxic T lymphocyte antigen 4 (CTLA4) are the most well characterized costimulatory and inhibitory molecules, respectively (159). Both are present on T cells, while their ligands CD80 (B7-1) and CD86 (B7-2) are expressed primarily on APCs (160). *CD28* mRNA was increased during cGvHD in PBMCs of patients (96). Interestingly, SNPs in *CTLA4* could have an impact on its function. In patients, the presence of the *A* allele in both rs231775 and rs3087243 was associated with a reduced risk of aGvHD after HSCT (161). Another study showed that recipients with the +49*A*/*G* allele had a significantly lower disease-free survival and overall survival in comparison to recipients with the *A*/*A* genotype (162). In addition to the +49*A*/*G* polymorphism, −1722, −1661, −318 polymorphisms in *CTLA4* were also evaluated after allo-HSCT, and a significant association between *GA* genotype (*CTLA4* −1661) and GvHD was shown in males with GvHD compared to males without GvHD (163). In addition, inducible T cell costimulator (ICOS), a member of the CTLA4 family that is expressed on activated T cells, was shown to be associated with the occurrence of aGvHD (19). The exact role of ICOS in GvHD is not clear; however, *ICOS* mRNA was downregulated in aGvHD patients. In contrast, *ICOS* mRNA was elevated during cGvHD in activated T cells in canines (164). Furthermore, blockade of ICOS *in vitro* during mixed lymphocyte reaction (MLR) resulted in immunosuppression, suggesting that ICOS plays a role in graft rejection and blockade of ICOS could be a potential therapeutic strategy (164). Cuzzola and colleagues also observed an increase in mRNA expression of *ICOS* in patients responding to anti-GvHD therapies as well as other genes that are regulated by ICOS, including Th2 cytokines (*IL4, STAT6*, and *IL18*) (19).

In addition to gene and miRNA expression studies in T cells, the characterization of the T cell receptor (TCR) repertoire in patients who underwent HSCT might be informative to assess risks of GvHD or relapse. It has been reported recently that these complications were associated with a lower TCR repertoire and the expansion of certain T cell clones (165).

### Gene Expression Profiles in B Cells Associated with Outcome of HSCT

B cells have been found to be important in contributing to cGvHD; however, the mechanisms involved in maintaining their activation are not known (166). Depletion of B cells reduced the incidence of cGvHD in mice (167). Elevated B cell-activating factor (*BAFF*), also known as tumor necrosis factor superfamily member 13b (*TNFSF13B*), mRNA levels were observed in patients with cGvHD and correlated to B cell activation (168). *BAFF* mRNA expression was also significantly upregulated in clinical GvHD patient biopsies in comparison to those with no GvHD (143). A differential pattern for gene expression for several genes was observed in the purified B cells from cGvHD patients on comparison with the B cells from healthy counterparts. Four of the genes, *IL12A*, interferon regulatory factor 4 (*IRF4*), *CD40*, and interferon gamma receptor 2 (*IFNGR2*), were downregulated whereas B cell linker (*BLNK*) mRNA was upregulated in B cells in patients with cGvHD (168). BLNK has been found to be important in proliferation and survival of B lymphocytes (169).

## Genome-Wide Association Studies (GWAS) for HSCT Outcome

**Figure 6:**
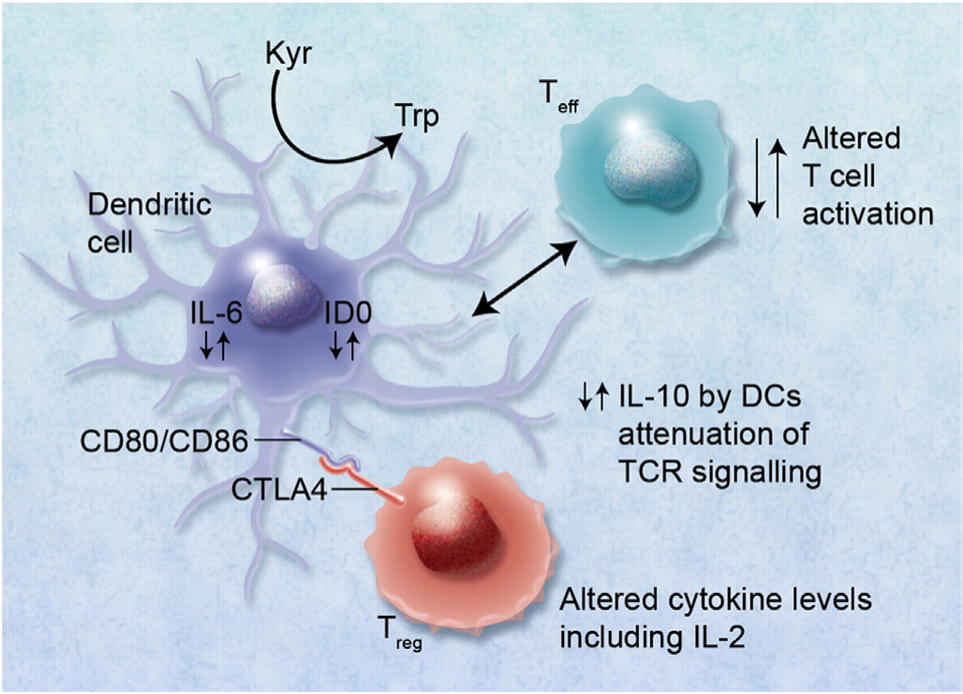
**Alterations in cytokine levels, such as interleukin (IL)-10, IL-6, and IL-2, *via* immunoregulatory single-nucleotide polymorphisms (SNPs) can lead to altered immunoregulatory function of regulatory T cells (T_regs_) and effector T cells (T_eff_)**. Binding of cytotoxic T lymphocyte antigen 4 (CTLA4) with its receptor (possibly also *via* functional single-nucleotide polymorphisms) with CD80/CD86 proteins on dendritic cells (DCs) can lead to induction of indoleamine 2,3 dioxygenase (IDO) and the catabolism of tryptophan into proapoptotic metabolites causing immunosuppression of T_eff_. Altered binding of CTLA4 may also lead to reduced immunosuppression *via* T_regs_ and GvHD. High IL-6 levels induced in DCs by T_reg_ interaction can also cause alteration of T_regs_ to Th17 cells and may lead to exacerbation of GvHD. The figure has been taken from Ref. (170). Professional illustration by Alice Y. Chen.

As a result of the Human Genome and the International HapMap Projects in the early 2000s (4, 5), GWAS became possible, expanding dramatically our capacity to understand genetic variability. A GWAS study of non-HLA SNPs in allogeneic HSCT was reported, which identified a number of SNP genotypes associated with severe aGvHD using a cohort of 1,298 patient donor pairs (139). The *IL6* donor genotype for rs1800795 was confirmed to be associated an increased risk of severe aGvHD. In addition other genes associated with aGvHD, *IL2*, methylene tetrahydrofolate reductase (*MTHFR*), Heparanase (*HPSE*), and cytotoxic T-lymphocyte-associated protein 4 (*CTLA4*) were identified in this GWAS cohort and illustrate (Figure 6) the fact that genomic control of immunoregulatory cytokines could alter the function of cells which in turn aid or reduce successful transplant outcome (170).

The Ogawa group has performed GWAS study in large cohort involving 1,589 patients and donors to identify miHAg associated with aGvHD (171). They identified three new loci that were significantly associated with severe (grade II–IV) aGvHD including the SNP rs17473423 within the *KRAS* locus. In a further GWAS study on a smaller identification cohort of 68 patients and a validation cohort of 100 patients, two GvHD susceptibility loci (rs17114803 and rs17114808) within the “suppressor of fused homolog” (*SUFU*) gene have been found (172). The incidence of aGvHD was significantly higher in patients that were homozygous for *CC* at SUFU rs17114808, than in heterozygous patients. Functional studies showed that ectopic expression of SUFU in DCs reduced expression of HLA-DR and suppression of MLR, whereas an increased HLA-DR expression and enhanced MLR was observed on silencing of SUFU (172). In the future, GWAS studies in HSCT will require larger multicenter cohorts but are expected to reveal new genetic associations for HSCT outcomes (8, 147).

## Problems with Gene Association Studies in HSCT

Genetic association studies have inherent difficulties when it comes to validation of results. Only robust genetic markers are able to be validated across HSCT cohorts. This is due to heterogeneity of transplants with regards to diagnosis, conditioning regimens, type of stem cells (e.g., peripheral blood, cord blood, or bone marrow), sibling or matched unrelated transplants and risk factors for outcome (CMV positivity; female to male donors, age of the transplant cohort) all of which alter the biology of the transplant itself. Problems associated with non-HLA genomics in HSCT have also been recently reviewed (8).

One example of this is within our own studies on SNP polymorphisms as risk factors for survival in chronic myeloid leukemia (CML). In the first study, presence of interleukin 1 receptor antagonist (*IL1RN*) allele 2 genotype in the donor (indicating downregulation of IL-1), absence of donor *IL10 ATA*/*ACC* genotype (indicating more downregulation of IL-10) and absence of tumor necrosis factor superfamily member 1B (*TNFSF1B*) 196R in the patient (indicating increased levels of soluble TNF-RII and decreased levels of TNF-α), all were associated with decreased survival and increased transplant relate mortality (173). In a validation cohort of matched unrelated transplants, none of the SNPs could be validated, and a comparison of the cohorts demonstrated differences in survival and clinical characteristics (174).

In addition, in a larger heterogeneous cohort, including CML and lymphoma (2), we developed a clinical and genetic score, which included the European Bone Marrow Transplantation (EBMT) Group score. This score incorporates clinical risk factors such as age of the patient and donor; time to transplant and type of transplant (175–178). Using a statistical analysis that included a bootstrap estimate of prediction error (179, 180), three further SNPs were associated with survival. A protective effect for the *IL10* genotype *ACC*/*ACC* in the donors was observed, while estrogen receptor 1 (*ESR1*) rs9340799 in the patient, *IL6* rs18000795 in the donors, and *TIRAP* (or *MAL*) rs177374 in the patient were associated with poorer survival (2). The subsequent clinical and genetic score assigned to each patient was shown to have a better predictive value than the EBMT score alone. In a more recent cohort studying acute leukemia transplant patients (181), three polymorphisms, presence of toll-interleukin 1 receptor domain containing adaptor protein (*TIRAP*) (alternatively named *MAL*) allele *T* (rs8177374) in the patient, absence of the glucocorticoid receptor (*GCR*) haplotype (consisting of rs6198, rs33389, and rs33388) *ACT* in the patient and absence of *HSPA1L* (or *HSP70-hom*) +2437 (rs2227956) allele *C* in the patient were associated with decreased survival. The subsequent clinical and genetic score assigned to each patient was shown to have a better predictive value than the EBMT score alone.

Interestingly, in all cohorts, the SNPs associated with reduced survival were all involved in downregulating the immune response, suggesting that this downregulation may be linked to reduced GvL responses and therefore lower overall survival.

These studies indicate that although replication of the exact genomic profiles may be difficult to achieve, the overall influence of genomics on the biology of the transplant is comparable and leads to a similar outcome, demonstrating that genomic studies are important for understanding the overall biology of the transplant.

## Conclusion

In this review, we have shown the differential expression patterns of a variety of mRNA, different miRNAs and SNPs in specific genes that have a significant impact on transplant outcome and development of GvHD. In addition, several SNP–mRNA–miRNA regulatory networks have been found to contribute to post-HSCT outcomes. Taken together, these findings demonstrate how expression of specific miRNAs can target the genes of key immune response modulators, directly affecting expression of mRNAs that influence the aGvHD response. However, mRNA and miRNA expression studies have not yet revealed a set of genes or miRNAs that can be used as reliable biomarkers for predicting the outcome of HSCT across different transplantation centers. Further, preferably multicentre, studies are required to determine SNPs and to analyze miRNA and mRNA expression in parallel in cohorts of HSCT patients to further elucidate genetic risks of HSCT. Such combined approaches have the potential to improve clinical practise of HSCT and eventually to benefit patients.

## Author Contributions

RG and PS drafted the manuscript; RC and JN commented and edited the draft; AD and RD supervised and revised the manuscript; and all authors approved the final version.

## Conflict of Interest Statement

The authors declare that the research was conducted in the absence of any commercial or financial relationships that could be construed as a potential conflict of interest. The reviewer RC and handling editor declared their shared affiliation, and the handling editor states that the process nevertheless met the standards of a fair and objective review.
